# Correction

**Published:** 1883-09

**Authors:** 


CORRECTION.

Last month we printed articles from two of our most esteemed exchanges without the appearance of the proper credit. We are sure that it was appended in the "copy," but it dropped off somewhere in the printing office. Our readers will therefore take notice that the extract in the August number headed "Facial Neuralgia," was from the July number of "THE AMERICAN JOURNAL OF THE MEDICAL SCIENCES"; and one entitled "Dropsy of the Antrum"

was taken from the "THE CANADA MEDICAL AND SURGICAL JOURNAL."

